# Pituitary Adenylate-Cyclase Activating Polypeptide Regulates Hunger- and Palatability-Induced Binge Eating

**DOI:** 10.3389/fnins.2016.00383

**Published:** 2016-08-22

**Authors:** Matthew M. Hurley, Brian Maunze, Megan E. Block, Mogen M. Frenkel, Michael J. Reilly, Eugene Kim, Yao Chen, Yan Li, David A. Baker, Qing-Song Liu, SuJean Choi

**Affiliations:** ^1^Department of Biomedical Sciences, Marquette UniversityMilwaukee, WI, USA; ^2^Department of Pharmacology and Toxicology, Medical College of WisconsinMilwaukee, WI, USA

**Keywords:** hypothalamus, accumbens, obesity, hedonic, homeostatic

## Abstract

While pituitary adenylate cyclase activating polypeptide (PACAP) signaling in the hypothalamic ventromedial nuclei (VMN) has been shown to regulate feeding, a challenge in unmasking a role for this peptide in obesity is that excess feeding can involve numerous mechanisms including homeostatic (hunger) and hedonic-related (palatability) drives. In these studies, we first isolated distinct feeding drives by developing a novel model of binge behavior in which homeostatic-driven feeding was temporally separated from feeding driven by food palatability. We found that stimulation of the VMN, achieved by local microinjections of AMPA, decreased standard chow consumption in food-restricted rats (e.g., homeostatic feeding); surprisingly, this manipulation failed to alter palatable food consumption in satiated rats (e.g., hedonic feeding). In contrast, inhibition of the nucleus accumbens (NAc), through local microinjections of GABA receptor agonists baclofen and muscimol, decreased hedonic feeding without altering homeostatic feeding. PACAP microinjections produced the site-specific changes in synaptic transmission needed to decrease feeding via VMN or NAc circuitry. PACAP into the NAc mimicked the actions of GABA agonists by reducing hedonic feeding without altering homeostatic feeding. In contrast, PACAP into the VMN mimicked the actions of AMPA by decreasing homeostatic feeding without affecting hedonic feeding. Slice electrophysiology recordings verified PACAP excitation of VMN neurons and inhibition of NAc neurons. These data suggest that the VMN and NAc regulate distinct circuits giving rise to unique feeding drives, but that both can be regulated by the neuropeptide PACAP to potentially curb excessive eating stemming from either drive.

## Introduction

A fundamental barrier in treating obesity is the challenge associated with isolating individual feeding drives. Understanding these could lead to the identification and development of potential new treatments based on the mechanisms underlying each unique form of caloric intake. Discrete forms of obesity, binge eating, or other eating disorders may differentially stem from pathological changes in circuitry underlying feeding typically driven by homeostatic needs (e.g., hunger-driven feeding) or hedonic motivations for highly palatable foods (e.g., palatable-driven feeding) (Lowe and Levine, 2005; Lowe and Butryn, 2007). However, the degree to which potential anorexigenic substances can suppress distinct feeding drives has been difficult to determine because feeding in many preclinical models likely involves multiple feeding drives. This is particularly problematic with paradigms comparing the consumption of standard chow and highly-palatable food in combination with food deprivation. For example, in the limited-access binge model, subjects have *ad lib* access to standard chow in conjunction with brief access to a highly palatable food, which promotes binge eating (Corwin, 2004; Corwin and Hajnal, 2005; Czyzyk et al., 2010). While *ad lib* access to standard chow should limit hunger-driven feeding, animals show self-induced food deprivation with reduced consumption of the devalued standard chow (Corwin and Buda-Levin, 2004). The potential that a confluence of homeostatic and hedonic drives exists in this model is evident by the observation that daily caloric intake and body weight remain stable in this paradigm despite the addition of the high-caloric food (Bake et al., 2014). In the current studies, we modified this approach by restricting access to both diets in order to promote conditions whereby hunger-driven consumption of standard chow resulted in satiety prior to providing subjects access to highly palatable food. By doing this, homeostatic and hedonic drives are more clearly separated, which enabled us to examine the cellular and molecular components of each feeding drive.

Using this new model of binge eating, we first sought to characterize the cellular or regional contributions to hunger- and palatable-driven feeding. Initially, we examined the impact of VMN activation on feeding primarily driven by homeostatic or hedonic feeding drives. Although the VMN have historically been viewed as satiety centers regulating feeding behavior (King, 2006), it is unknown if the VMN-satiety signal also gates feeding stemming from other distinct drives (e.g., palatable-driven feeding). We then targeted subregions of the nucleus accumbens (NAc), which has been principally linked to hedonic drives; the degree to which the NAc regulates other motivations to eat including homeostatic-based feeding is less well studied (Baldo and Kelley, 2007; Johnson and Kenny, 2010; Baldo et al., 2013). Each of these experiments is important because human obesity can stem from either abnormal homeostatic feeding or, over consumption of highly palatable foods even in the absence of homeostatic need (Boggiano, 2016). Hence, these and future studies have the potential to identify drive-specific circuitry, a discovery that could help narrow attempts to outline the neural basis for unique forms of obesity.

An additional objective was to examine the potential for a single anorexigenic substance to modify the activity of both NAc- and VMN-related circuitry through either hunger- or palatable-feeding drives. Recently, we found that intra-VMN administration of pituitary adenylate cyclase-activating polypeptide (PACAP) markedly suppressed feeding and reduced body weight even in fasted animals via the PAC1R receptor subtype (Resch et al., 2011, 2013). Of the three PACAP receptors, PAC1R is primarily involved in the hypophagic properties of intra-VMN PACAP whereas the contribution of VPAC1 and VPAC2 are not (Resch et al., 2013). While the VMN express an abundant amount of PACAP mRNA, retrograde tracing has revealed numerous extra-hypothalamic efferents including PACAP containing projections from the medial amygdala and lateral parabrachial (Resch et al., 2013). In the NAc, similar retrograde studies show different PACAP containing efferent projections to the NAc such as the medial prefrontal cortex (unpublished data). PACAP is a highly conserved neuropeptide that is often expressed in glutamatergic neurons and has been primarily implicated in neurohormone signaling, learning and memory, and neurodegenerative responses (Pellegri et al., 1998; Zhou et al., 2002). Thus, it represents an interesting molecular candidate because prior studies have shown that this neuropeptide is capable of activating and inhibiting ionotropic glutamate receptors (Macdonald et al., 2005; Toda and Huganir, 2015). For example, PACAP's anorexic actions in VMN likely augments glutamate signaling by potentiating NMDA receptors (Resch et al., 2014b). Hence, the capacity for PACAP to produce bidirectional changes in excitatory signaling may position this poorly understood anorexigenic peptide to inhibit NAc-related circuitry and suppress palatable-driven feeding while stimulating VMN-related circuitry to restrict hunger-driven feeding.

## Materials and methods

### Animals

Male Sprague-Dawley rats (Harlan; Indianapolis, IN) weighing 350–400 g, were housed individually in either a BioDAQ feeding system, a computer automated data acquisition system that records food intake measurements using an algorithmic load cell technology (Research Diets, New Brunswick, NJ) or standard hanging wire cages under a 12:12 light/dark cycle. Feeding was measured via the BioDAQ system or by weighing food bins before and after experimental sessions (including spilled food). Body weights were collected daily. All animal procedures were approved by the Marquette University Institutional Animal Care and Use Committee.

### Diets

We used Harlan standard chow (SC; #8604; 32% protein, 54% carbohydrate, 14% fat; 3.0 kcal/g) or a palatable western diet (WD; #D12079B; Research Diets; New Brunswick, NJ; 17% protein, 43% carbohydrate, 41% fat; 4.7 kcal/g). When indicated, standard chow was flavored with either vanilla, almond (0.05% pure vanilla extract, 0.05% imitation almond extract; The J.R. Watkins Co; Winona, MN) or vehicle (water).

### Cannulation surgery and microinjections

#### Surgery

Animals were anesthetized with ketamine/xylazine/acepromazine (77:1.5:1.5 mg/ml/kg; i.p.). Twenty-six-gauge bilateral guide cannulae (Plastics One; Roanoke VA) were stereotaxically placed 2–3 mm above the ventromedial nuclei (VMN; anterior/posterior, −2.5 mm from bregma; medial/lateral, ± 0.6 mm from midline; dorsal/ventral, −6.2 mm from surface of the skull) or the nucleus accumbens (NAc; anterior/posterior, +1.6 mm from bregma; medial/lateral, +2.2 from midline; dorsal/ventral, −4.8 mm from surface of the skull) and secured to the surface of the skull (Paxinos and Watson, 2007). Afterwards, brains were collected, immediately frozen and embedded in OCT for analysis of cannula placement. Thirty micrometers thick sections were Nissl stained and only those with correct placements were included in the studies (**Figure 5**).

#### Microinjections

Pituitary adenylate cyclase activating polypeptide (PACAP; 50 pmol/0.25 μl/side; California Peptide Research, Napa, CA), α-amino-3-hydroxy-5-methyl-4-isoxazolepropionic acid (AMPA; 74.5 ng/side; Tocris Bioscience, Minneapolis, MN); baclofen+muscimol (106.8 ng/5.7 ng/side; Tocris Bioscience, Minneapolis, MN) or saline (vehicle) were microinjected into the VMN (0.25 μl/side) or NAc (0.5 μl/side) over a 2 min period (using a syringe pump) in gently restrained awake animals followed by an additional minute to prevent backflow.

### Restricted feeding

At the onset of dark, animals (*n* = 12 total) were entrained (1 week/regimen) to various restricted feeding durations (2, 3, or 4 h/day in BioDAQ) using only SC. During the remaining 22, 21, or 20 h animals did not have access to food. Body weights were recorded daily. In addition to the restricted feeding groups, animals (*n* = 6/group) fed SC and WD *ad libitum* served as control groups for feeding and body weight measurements.

### Two-meal model (M1-M2)

Rats (*n* = 12/group) were entrained to consume their daily SC intake in a 2-h period after the onset of the dark phase (Meal 1; M1). After establishing consistent feeding patterns and weight gain (40–50 kcal/2 h; body weight gain 2–3 g/day), animals were offered a short 15 min meal (Meal 2; M2) of either SC or WD (*n* = 6/group) ~30 min following M1 for 7 days before experimentation. Food intake and body weight measurements were recorded in an additional group (*n* = 6/group) of rats that were *ad lib* fed either SC or WD as additional control groups.

In separate studies, animals were entrained to the two-meal model (M1-M2) for 5 days before undergoing VMN or NAc cannulation surgery. VMN microinjections of vehicle (*n* = 9–10/group), PACAP (*n* = 7/group), AMPA (*n* = 6/group), or baclofen+muscimol (*n* = 3/group) were separately administered ~30 min prior to either M1 or M2. Similarly, NAc microinjections of vehicle (*n* = 9–10/group), PACAP (*n* = 9/group), baclofen+muscimol (*n* = 9/group), or AMPA (*n* = 3/group) were administered ~30 min prior to M1 or M2.

### Slice electrophysiology

Rats were anesthetized by isoflurane inhalation and decapitated. Coronal slices (250 μm; *n* = 6–7 slices/brain region) containing the VMN and the NAc were cut using a vibrating slicer (VT1000S, Leica) at 4°C with a sucrose-based solution containing the following: 220 mM sucrose, 25 mM NaHCO_3_, 2.5 mM KCl, 1.25 mM NaH_2_PO_4_, 0.5 mM CaCl_2_, 7 mM MgSO_4_, and 10 mM glucose. The slices were recovered in a sucrose-NaCl-based solution containing the following: 68 mM sucrose, 78 mM NaCl, 25 mM NaHCO_3_, 2.5 mM KCl, 1.25 mM NaH_2_PO_4_, 2 mM CaCl_2_, 1 mM MgCl_2_, and 10 mM glucose for 30 min at room temperature. The slices were then transferred to artificial cerebrospinal fluid (ACSF) containing the following: 125 mM NaCl, 2.5 mM KCl, 2.5 mM CaCl_2_, 1 mM MgCl_2_, 1.25 mM NaH_2_PO_4_, 25 mM NaHCO_3_, and 10 mM glucose. The slices were maintained in ACSF for at least 1 h before electrophysiology recordings. All solutions are saturated with 95% O_2_ and 5% CO_2_.

Whole-cell or cell-attached recordings were made from the VMN and NAc using patch-clamp amplifier Multiclamp 700B under infrared-differential interference contrast (DIC) microscopy. The VMN is an egg-shaped region located in the mediobasal hypothalamus adjacent to the third ventricle, and the NAc is an area around the optic nerve about 200 μm from the edge of the anterior commissure. Data acquisition was performed using DigiData 1440A digitizer (Molecular Devices). Glass pipettes (4–6 MΩ) were filled with an internal solution containing (in mM): 140 potassium gluconate, 5 KCl, 10 HEPES, 2 MgCl_2_, 0.2 EGTA, 2 MgATP, 0.3 Na_2_GTP, and 10 Na_2_-phosphocreatine (pH 7.4 with KOH). Signals were filtered at 2 kHz and sampled at 10 kHz. Spikes were driven by current injections from −60 to 300 pA. PACAP (100 nM) was added to the brain slices after the membrane potential was stable and a baseline measurement (control) of spontaneous activity and spike firing followed by application of PACAP to obtain within cell treatment effects. Glutamate receptor antagonist CNQX (10 μM) and GABA_A_ receptor blocker picrotoxin (50 μM) were present throughout all physiological recordings. Recordings were performed at 32 ± 1°C using an automatic temperature controller (Warner Instrument).

### Corticosterone (B) radioimmunoassay

In a separate group of animals offered *ad lib* SC (*n* = 12) or restricted SC access (2 h/day at the onset of the dark cycle; *n* = 12) for 2 weeks, half were sacrificed at the onset of the dark cycle (prior to eating), and the remaining half sacrificed 2 h into the dark cycle or after the restrict feeding session. Plasma B was measured from trunk blood using a radioimmunoassay (MP Biomedicals, Santa Ana, CA).

### Statistics

Data are presented as means ± standard error of the mean and analyzed by ANOVA (with repeated measures when appropriate) or Student's *t*-test. Fisher LSD analysis was used for *post-hoc* group comparisons using Sigma Plot 11 software (Systat Software Inc.; San Jose, CA). *p* < 0.05 = statistical significance.

## Results

### Two-meal model (M1-M2) and restricted feeding

**Figure 1:**
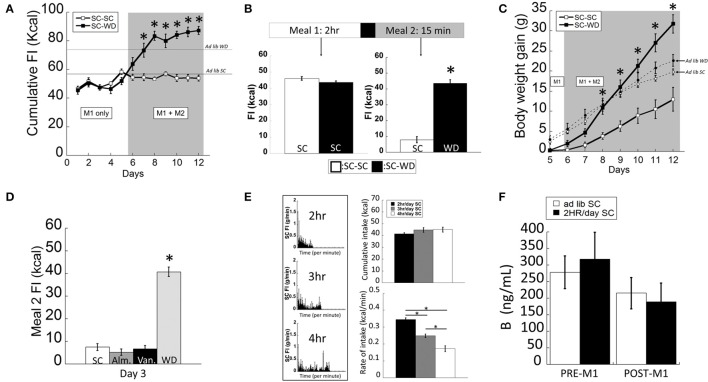
**The two-meal paradigm maintained animals on restricted SC (standard chow) intake (M1; 2 h/day) followed by access to a second meal (M2; 15 min/day) of SC (M1(SC)-M2(SC)) or a highly palatable diet (WD) shortly after M1, (M1(SC)-M2(WD))**. **(A)** SC-WD displayed higher total food intake (FI) levels than SC-SC animals (and *ad lib* fed SC or WD animals). **(B)** By day 12, satiated rats offered WD (SC-WD) consumed more calories than animals offered SC (SC-SC). **(C)** Animals consuming WD (SC-WD) during M2 gained significantly more weight than animals offered SC (SC-SC) and gained more than *ad lib* fed SC and WD animals. **(D)** Food intake levels of SC flavored with almond (Alm.) or vanilla (Van.) during M2 did no differ compared to unflavored SC and were significantly lower than WD. **(E)** Left: SC food intake (FI) levels for 2, 3, or 4 h daily access; Upper-right: cumulative daily SC intake did not differ between 2, 3, or 4 h; Lower-right: 2 h feeding periods resulted in significantly faster feeding rates compared to 3 or 4 h access. **(F)** Plasma B (corticosterone) levels in *ad lib* and restrict fed animals before M1 and after the onset of dark and 2 h into the dark cycle (after M1). Data expressed as mean ± SEM. ^*^p < 0.05.

The two-meal model tested food consumption in satiated vs. hungry rats. After entrainment to a 2 h SC meal (M1), animals were offered a second meal (M2; 15 min) consisting of either SC or WD (Figure 1A). Animals consuming WD during M2 (SC-WD) consumed significantly more total daily calories than rats receiving SC (SC-SC) [Figure 1A; DIET *F*_(1, 183)_ = 78.428, *p* < 0.001; DIET × TIME *F*_(7, 183)_ = 13.279, *p* < 0.001]. Moreover, Figure 1A shows SC-WD fed animals consumed more calories than animals provided SC (58.6 ± 0.5 Kcal) and WD *ad libitum* (74.9 ± 1.4 Kcal) demonstrating that 2 h restricted feeding or SC-SC resulted in ~25% reduction in daily caloric intake compared to a SC *ad lib* fed animals and that SC-WD animals consumed more calories than *ad lib* WD fed rats. By day 12, animals consumed as many calories from WD during the 15-min M2 as was consumed during the 2 h M1 (Figure 1B; *p* < 0.001) and as a result gained significantly more body weight than rats receiving SC for M2 [Figure 1C; DIET × TIME; *F*_(6, 150)_ = 32.983; *p* < 0.001]. Notably, this increase was significantly greater than even *ad lib* WD fed rats. A significant difference in body weight gain was evident by the third presentation of WD for M2 compared to the SC-SC group (*p* < 0.001; Figure 1C). In addition, SC-WD animals gained body weight faster than animals maintained on *ad lib* SC or WD (Figure 1C). To determine if the two-meal model was a product of novelty, we offered SC made novel by flavoring with either vanilla or almond extract (or control) during M2. There were no differences in M2 intake over 3 days access to flavored SC (Figure 1D; SC vs. almond *p* = 0.714; SC vs. vanilla *p* = 0.902; almond vs. vanilla *p* = 0.807), which contrasted the marked increase in palatable WD intake over the same time period (Figure 1D; WD vs. SC, almond or vanilla *p* < 0.001).

In order to develop a feeding paradigm that produced fully-satiated animals (i.e., minimal homeostatic-based feeding), we measured the total calories consumed during periods of 2–4 h of restricted feeding in 1 min bins (Figure 1E). Interestingly, total intake of SC did not differ in rats permitted 2, 3, or 4 h daily access [*F*_(2, 35)_ = 0.781; *p* = 0.466]. As would be predicted, animals provided 2 h access to SC ate at a faster rate (kcal/min) compared to rats allowed 3 or 4 h access [Figure 1E; *F*_(2, 35)_ = 76.749; *p* < 0.001]. Restricted feeding of SC at all durations was sufficient to produce a modest weight gain (data not shown). We and others have shown that animals entrained to 2 h of restricted feeding show normal circadian rhythmicity and low basal and normal peak levels of corticosterone (Figure 1B; <3 μg/dl and >20 μg/dl, respectively) suggesting that they were not chronically stressed (Krieger, 1980; Choi et al., 1998). In support, we confirmed that circulating B levels did not differ between *ad lib* and 2 h restrict fed animals (before and after their meal) during a period of peak B activity [Figure 1F; FEEDING REGIMEN *F*_(1, 23)_ = 0.017, *p* = 0.915; FEEDING REGIMEN × TIME *F*_(1, 23)_ = 0.302, *p* = 0.589]. Taken together, we chose the 2-h restricted feeding to ensure a state of satiety in the shortest amount of time.

### VMN microinjections

**Figure 2:**
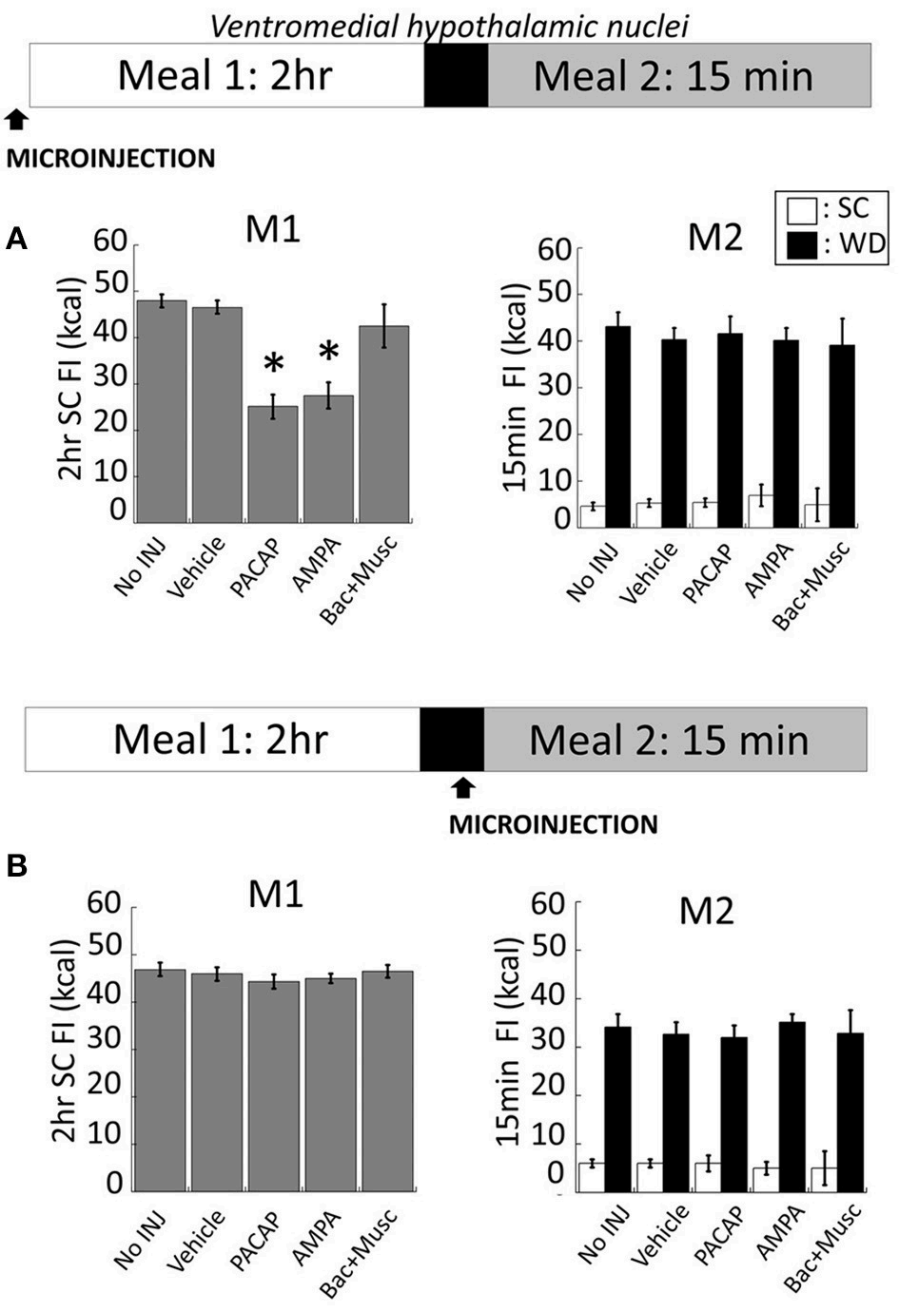
**PACAP or AMPA microinjections into the hypothalamic ventromedial nuclei (VMN) suppressed hunger-induced feeding (meal 1; M1) without affecting palatable food consumption (meal 2; M2)**. **(A)** Intra-VMN PACAP or AMPA administered prior to M1 significantly suppressed hunger-induced feeding of standard chow (SC) compared to baclofen+muscimol, vehicle (saline), and non-injected controls, whereas there were no significant treatment difference in the consumption of SC or palatable diet (WD) during M2. **(B)** No changes in SC or WD consumption following microinjections administered just prior to M2. Data expressed as mean ± SEM. ^*^p < 0.05.

Intra-VMN PACAP (**Figure 5A** for anatomy) administered prior to M1 produced a significant reduction in SC consumption during M1 compared to non-injected (No INJ) and vehicle injected animals (Figure 2A; *p* < 0.001 for both). Intra-VMN AMPA administration also significantly suppressed consumption of SC during M1 (Figure 2A; AMPA vs. No INJ or vehicle, *p* < 0.001; baclofen+muscimol vs. No INJ, *p* = 0.148; vs. vehicle, *p* = 0.282) indicating that PACAP and AMPA produced similar behavioral actions in the VMN. Surprisingly, there were no differences in the calories consumed during M2 of either SC or WD when PACAP was administered prior to M1 (Figure 2A; PACAP vs. No INJ, *p* = 0.624; vs. vehicle, *p* = 0.713) or just prior to M2 (Figure 2B; PACAP vs. No INJ, *p* = 0.613; vs. vehicle, *p* = 0.868). Baclofen+muscimol injections into the VMN did not alter feeding during either M1 or M2 suggesting that PACAP actions in the VMN are primarily excitatory. Every cannula placement into the VMN was confirmed at the conclusion of the study resulting in a 90% accuracy rate.

### NAc microinjections

**Figure 3:**
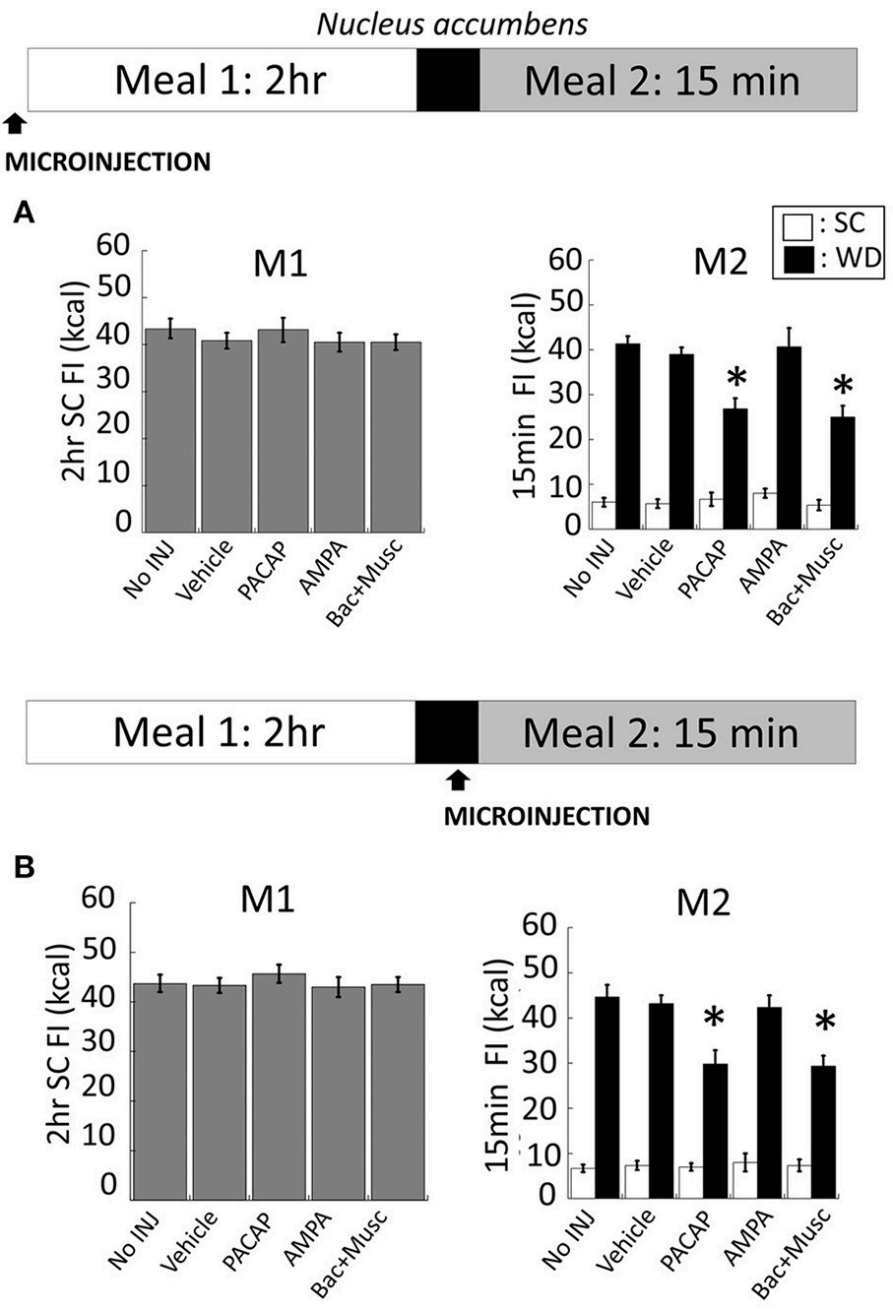
**PACAP and baclofen+muscimol microinjections into the nucleus accumbens (NAc) suppressed palatable food consumption (meal 2; M2) without effecting standard chow (SC) intake (meal 1; M1)**. **(A)** No changes to SC intake following microinjections administered prior to M1 compared to controls. However, intra-NAc PACAP or baclofen+muscimol significantly reduced WD intake compared to AMPA, vehicle and non-injected controls during M2. **(B)** Similarly, WD intake was reduced following PACAP and baclofen+muscimol administration into the NAc just prior to M2. Data expressed as mean ± SEM. ^*^p < 0.05.

NAc injections of PACAP (Figure 5B for anatomy), AMPA, or baclofen+muscimol (prior to M1) had no effect on feeding behavior during M1 [Figure 3A; *F*_(4, 80)_ = 0.463; *p* = 0.763]. However, intra-NAc injections of PACAP and baclofen+muscimol significantly reduced WD intake during the subsequent 15 min M2 compared to vehicle and non-injected controls (Figure 3A; PACAP vs. No INJ, *p* < 0.001; vs. vehicle, *p* < 0.002; baclofen+muscimol vs. No INJ or vehicle, *p* < 0.001). Similarly, PACAP and baclofen+muscimol administered just prior to M2 also suppressed WD intake (Figure 3B; PACAP vs. No INJ or vehicle, *p* < 0.001; baclofen+muscimol vs. No INJ or vehicle, *p* < 0.001). By contrast, AMPA administration into the NAc prior to either M1 or M2 had no effect on food consumption suggesting that PACAP actions in the NAc were inhibitory. Every cannula placement into the NAc was confirmed at the conclusion of the study resulting in a 90% accuracy rate.

### Slice electrophysiology

**Figure 4:**
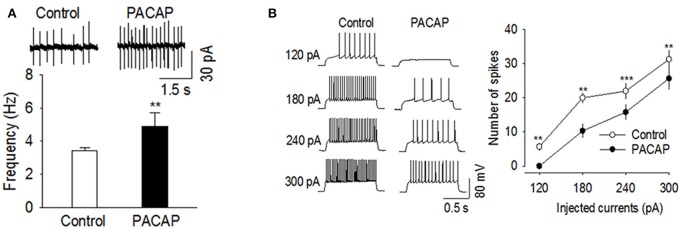
**PACAP produced opposite effects on action potential firing in the VMN and NAc. (A)** PACAP significantly increased the frequency of spontaneous action potential firing in the VMN (*n* = 7, ^**^p < 0.01 vs. control). **(B)** PACAP decreased the number of spikes in the NAc in response to depolarizing current injections (120–300 pA, *n* = 6, ^**^p < 0.01 vs. control, ^***^p < 0.001 vs. control, Paired *t*-test). All physiological recordings were collected in the presence of CNQX and picrotoxin.

We determined whether PACAP affected action potential firing rates in VMN and NAc slices. All recordings were made in the presence of the glutamate receptor antagonist CNQX (10 μm) and the GABA_A_ receptor blocker picrotoxin (50 μm) to block excitatory and inhibitory synaptic transmission. Cell-attached patch clamp recordings were made on VMN neurons, which displayed spontaneous action potential firing. Bath application of PACAP (100 nM) significantly increased the frequency of spontaneous action potential firing in VMN neurons [Figure 4A, *t*_(6)_ = −4.062, *n* = 7, *p* < 0.004, Paired *t*-test]. We next examined whether PACAP also affected action potential firing in the NAc. Since medium spiny neurons (MSNs) in NAc slices do not fire spontaneous action potentials at resting membrane potential (~−80 mV), we made whole-cell current-clamp recordings and evoked action potential firing by injecting depolarizing current steps. Bath application of PACAP (100 nM) significantly decreased the number of spikes in responses to depolarizing current injections [Figure 4B, 120 pA, *t*_(5)_ = 4.828, *p* < 0.005; 180 pA, *t*_(5)_ = 4.620, *p* < 0.006; 240 pA, *t*_(5)_ = 11.364, *p* < 0.001; 300 pA, *t*_(5)_ = 5.937, *p* < 0.002, *n* = 6]. These effects were independent of excitatory and inhibitory synaptic inputs as these studies were conducted in the presence of both CNQX and picrotoxin. Thus, PACAP increased spontaneous action potential firing in the VMN whereas, it decreased evoked action potential firing in the NAc.

**Figure 5:**
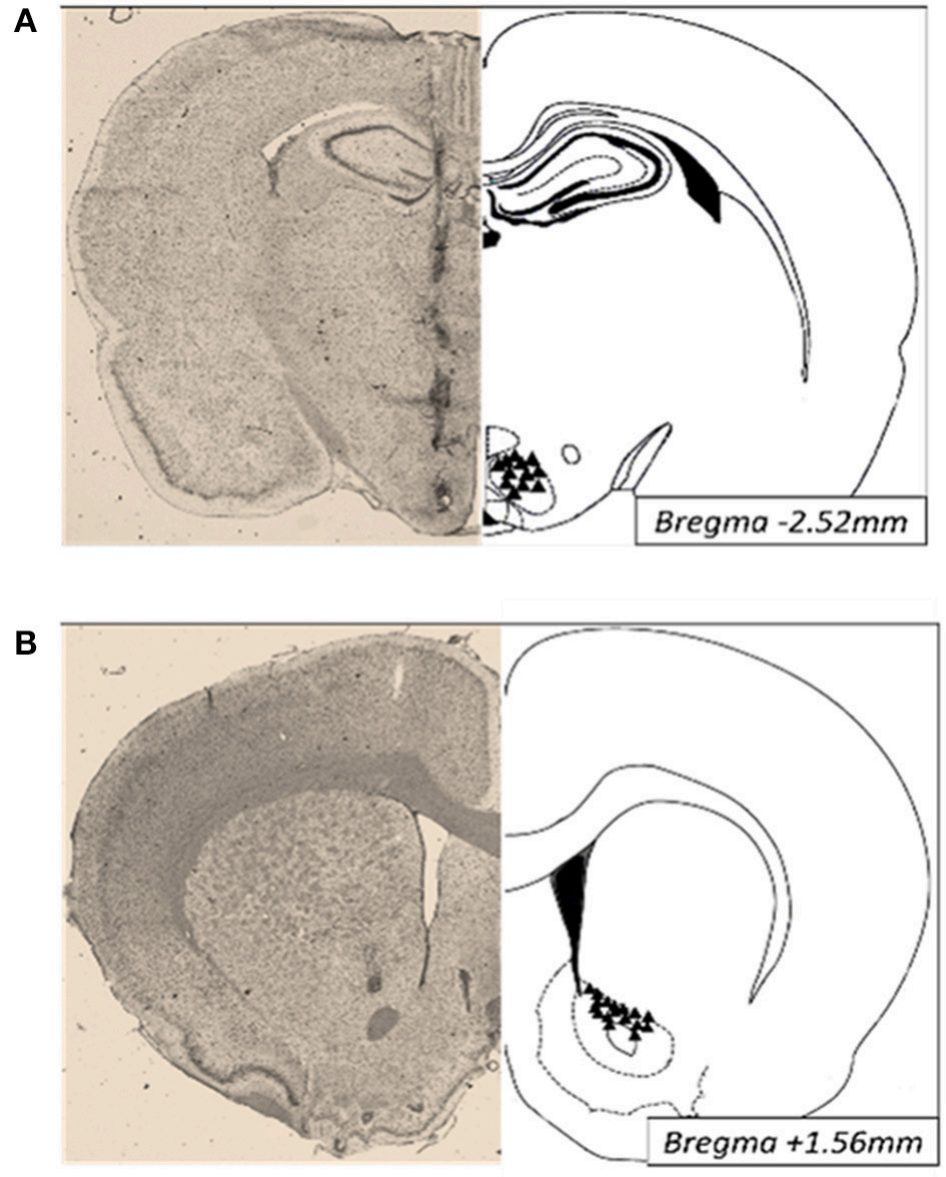
**Photomicrographs of cannula placements (triangles, right) and a representative Nissl stained section (30 μm; left) of the VMN (A) and NAc (B)**.

## Discussion

Obesity can stem from excessive or binge-like consumption of food generated by different homeostatic and hedonic-related drives, each of which may involve distinct circuitry in the brain. This study extends earlier findings revealing that PACAP administration into the hypothalamic VMN markedly suppressed feeding behavior (Resch et al., 2011, 2013) by determining the capacity of this novel anorexigenic peptide to regulate distinct forms of eating stemming from homeostatic and hedonic feeding drives. To do this, we developed a novel binge-eating paradigm (rapid consumption of a high volume of food within a short time period) that would better isolate distinct feeding drives. Using this paradigm, it is likely that VMN activation suppressed the consumption of standard chow (SC) in restrict-fed rats without altering palatable food intake in a satiated rat. Inhibition of the NAc produced the opposite outcome in that consumption of palatable food in a satiated rat was reduced, while SC intake was not altered. Interestingly, PACAP signaling in the VMN and NAc produced the precise changes in synaptic transmission needed to suppress each form of eating. Collectively, these data suggest that distinct feeding drives may involve at least partially non-overlapping circuitry, and that targeting PACAP signaling may be an effective strategy at reducing both homeostatic and hedonic-related feeding.

### Isolation of homeostatic- and hedonic-related feeding drives

A challenge in the study of the neurobiology of obesity is that multiple feeding drives are likely *simultaneously* activated under most experimental conditions thereby, obfuscating efforts to identify the cellular or molecular basis of discrete feeding drives (Lowe and Levine, 2005; Lowe and Butryn, 2007). Many rodent models assess consumption of a highly-palatable food combined with some degree of food deprivation, thereby demonstrating the presence of multiple feeding drives even in those designed to separate distinct drives. For example, in the limited-access binge model, rodents are provided *ad lib* access to SC and limited-access to palatable foods often high in both fat and sugar (Corwin, 2004; Corwin and Hajnal, 2005; Czyzyk et al., 2010). While *ad lib* SC intake should mitigate hunger-driven feeding during the limited access to a highly palatable diet, rats display self-imposed deprivation evident by significantly decreased SC consumption. Thus, both hunger- and palatability-driven feeding drives are likely engaged during the limited access period. In the current approach, we limited the co-existence of hunger-driven and palatability-driven feeding drives by creating conditions in which restricted-feeding produces heightened hunger-driven feeding that is satiated with a low-palatable diet. It is important to note that the study of the neural mechanisms underlying feeding involving *hunger*- or *palatability*-related drives requires the manipulation of these variables to understand the specific contribution of each of these drives. There are three key design aspects used to create this desired experimental condition. First, subjects were restrict fed for 2 h; these conditions do not result in overt increases in stress hormones (Choi et al., 1998) and has previously been used by numerous others to enhance hunger-driven feeding (Hagan and Moss, 1997; Denis et al., 2015; Wei et al., 2015; Baldo et al., 2016). Confounding interactions stemming from malnourishment in these animals is extremely unlikely since rats with similar long-term caloric restriction paradigms (25% reduction) show improved life expectancy and health outcomes (Keenan et al., 1996, 2013). Second, SC was used as the low-palatable diet, which is illustrated in other studies to show diminished motivation for SC after exposure to a palatable diet (South et al., 2014). Third, the duration of the 2-h restricted feeding of SC was sufficient to produce satiety as evident by the lack of increased consumption when the access period was lengthened to 3 or 4 h. Hence, these conditions permit the study of the cellular or molecular basis of hunger-driven eating that culminates in a robust state of satiety.

In the next phase of the paradigm, palatability-driven feeding was assessed by measuring feeding in satiated rats provided additional access (15 min) to either low- or high-palatable diets. As expected, palatable food consumption significantly increased compared to the minimal consumption of SC. Remarkably, the average number of calories consumed of a highly-palatable food by satiated rats (M2) was equivalent to the number of SC calories consumed during the 2-h M1. This marked increase in the highly palatable food is unlikely to be due to novelty or stimulus-specific satiety since a similar increase was not obtained when SC was made novel with either vanilla or almond flavorings (Figure 1D). Hence, these conditions likely permit the study of the cellular or molecular basis of palatable-driven feeding with limited influence from hunger-driven eating.

### VMN gate hunger but not palatability-driven feeding

Historically, the VMN were thought to be critical components of the brain's “satiety center” (Kennedy, 1950) and later described as the inhibitory counterpart to the lateral hypothalamus (promoting feeding) in the dual-center hypothesis for motivated behavior (Stellar, 1954). Recent studies continue to support the VMN as key sites in the regulation of energy homeostasis by demonstrating that specific genetic deletions in the VMN lead to obesity (Kim et al., 2009), altered fMRI activity is evident in the VMN after ingesting a glucose solution (Liu and Gold, 2003), and a positive correlation between the degree of medial hypothalamic damage and excess weight gain (Pinkney et al., 2002). However, an important outstanding question is whether the satiety signal from the VMN regulates multiple distinct feeding drives (e.g., hunger and palatability-driven feeding). Using the two-meal paradigm, we found that VMN activation achieved by local AMPA injections decreased consumption of SC in restrict-fed rats but, surprisingly, it did not alter palatability-driven feeding. While more work is needed to more thoroughly characterize this effect, these findings are consistent with the conclusion that hunger- and palatability-driven feeding involve at least partially non-overlapping circuitry.

### NAc gates palatability but not hunger-driven feeding

The NAc has been strongly implicated in a wide-range of motivated behaviors, including palatability-driven feeding (Robbins and Everitt, 1996; Wise, 1998; Aragona et al., 2006; Baldo and Kelley, 2007). However, an open question is whether NAc-related circuitry are also involved in hunger-driven feeding, in part because many studies measure intake when both hunger- and palatability-related drives would be present. We found that local inactivation of the NAc by baclofen+muscimol reduced palatability-driven but not hunger-driven feeding. Our finding that GABA agonists into the NAc did not reduce hunger-driven consumption of SC is consistent with earlier work (Stratford and Kelley, 1997). However, we are the first to show that inhibition of the NAc decreased hedonic-driven feeding in rats that were accustomed to binge eating a palatable meal. While it is possible that regions of the NAc or ventral striatum not impacted by our manipulations may contribute to both forms of eating, our results, at a minimum, reinforce the concept that each of these feeding drives can involve unique circuitry. Illustrating this point is the evidence that GABA agonist administration in other regions of the NAc show increased feeding behavior (Basso and Kelley, 1999). Thus, discretely mapping the anatomical underpinnings of various feeding drives could provide key insight into the etiology of eating behavior underlying distinct forms of obesity. For example, individuals displaying excess eating stemming from enhanced hunger-driven feeding vs. those that display enhanced (or the inability to suppress) palatable-driven feeding may express unique molecular and cellular pathological changes that could be targeted by more focused therapeutic intervention.

### PACAP gates both hunger- and palatability-driven feeding

In the NAc, microinjections of PACAP did not alter homeostatic feeding but effectively reduced consumption of a highly palatable diet. Specifically, intra-NAc PACAP only altered consumption of high-fat, high-carb food in a satiated rat. The lack of an effect on homeostatic feeding is unlikely to be due to an insufficient dose or drug duration given that identical parameters were used in the VMN to block homeostatic feeding and in the NAc to block palatable feeding. Interestingly, the activation of NAc efferents, all of which are GABAergic, is linked to multiple forms of motivated behavior including palatability-driven feeding, as described above (Robbins and Everitt, 1996; Wise, 1998; Aragona et al., 2006; Baldo and Kelley, 2007). Thus, our observation that PACAP in the NAc mimicked the behavioral effects of GABA agonists suggests that PACAP likely inhibited at least some of these circuits, although as discussed below, the precise mechanism is unknown.

In the VMN, we found that microinjections of PACAP reduced homeostatic but not hedonic feeding. In support, PACAP microinjections into the VMN decreased consumption only when rats displayed a pronounced hunger drive (e.g., following a 22 h fast). Once the animal achieved a state of satiety, PACAP microinjections into the VMN did not alter the consumption of either standard chow or a highly palatable food source. Interestingly, PACAP in the VMN mimicked the actions of AMPA microinjected into this structure. Given that previous studies have established the VMN as a satiety center of the brain in which activation of this structure reliably decreases feeding, these collective results suggest that both PACAP and AMPA excited VMN efferents involved with satiety. While our experiments did not identify the type of cell impacted by PACAP, previous studies have revealed that the majority of VMN cells are glutamatergic (Bowers et al., 1998; Ovesjö et al., 2001). In support, studies have shown highly dense expression of the glutamatergic marker vGlut2 (Ziegler et al., 2002) with minimal expression of non-glutamatergic cells.

Our finding that PACAP signaling in the VMN reduces homeostatic but not hedonic feeding extends existing work establishing the hypophagic and metabolic actions of this neuropeptide. Although PACAP signaling has been implicated in feeding behavior and body weight regulation for over 20 years (Morley et al., 1992; Chance et al., 1995), only recent studies have begun to delineate its regional and mechanistic details. PACAP administration into the VMN reduces *ad lib* feeding without malaise specifically through the PAC1R receptor subtype, while also increasing thermogenesis and spontaneous locomotor activity (Resch et al., 2011). Likely as a result of both the anorexia and the increased metabolic indices, PACAP in the VMN results in dramatic body weight loss even after a single acute administration (Resch et al., 2011, 2013). Moreover, PACAP administration in the VMN increases both POMC mRNA expression in the arcuate nuclei and fasting glucose levels further illustrating a role for PACAP in the regulation of energy balance.

Given the historical roles for the NAc in generating motivated behaviors and the VMN in suppressing feeding, it would seem that a molecule acting in each structure would need to have the remarkable capability of inhibiting the NAc while activating the VMN to regulate each form of eating. While more work needs to be done to confirm these effects for PACAP, our data are consistent with this type of region-specific regulation. In the current study, we found that bath application of PACAP to VMN slices increased action potential firing and that microinjections of PACAP and AMPA produced the same behavioral effect. In the NAc, PACAP appears to produce the opposite effect in that bath application of PACAP to NAc slices decreased evoked potentials and microinjections of PACAP into the NAc mimicked the effects of baclofen+muscimol on feeding.

While the current results do not identify the molecular basis for PACAP mimicking GABA agonists in the NAc and AMPA in the VMN, previous work has shown that PACAP is able to increase or decrease the activity of glutamate ionotropic receptors, including NMDA (Shioda et al., 1997; Vaudry et al., 2009; Toda and Huganir, 2015). Lastly, previous work has also linked PACAP to other glutamatergic mechanisms, such as system x${}_{\text{c}}^{-}$ (Resch et al., 2014a; Kong et al., 2016) and activation of metabotropic glutamate receptors (Baker et al., 2002, 2003), which may display region-specific differences in expression (Gu et al., 2008). Regardless, these data show the degree to which the complexity of the glutamate network can differ across discrete brain regions yet be regulated by the same neuropeptide, potentially revealing PACAP to be a powerful regulator of caloric intake by both activating or inhibiting circuits associated with satiety (e.g., VMN) and appetitive (e.g., NAc) signals, respectively. Future studies will be needed to explore this intriguing possibility.

Collectively, these data suggest that PACAP signaling suppresses multiple feeding drives, which positions this novel anorexigenic peptide as an important target in understanding and possibly treating obesity. Toward the latter observation, identifying therapeutic targets capable of modulating multiple feeding drives may be especially important in the treatment of obesity given the widely observed propensity for tolerance to anti-obesity medications to have long-term utility (Fernstrom and Choi, 2008), an effect that could be due to compensatory changes across distinct drives. Thus, these findings may address a fundamental barrier in treating obesity by better isolating individual feeding drives and demonstrating the potential for PACAP signaling to regulate unique forms of overeating.

## Author contributions

MH, DB, QL, and SC designed research; MH, BM, MB, MF, MR, EK, YC, and YL performed research; MH, MF, MR, YC, YL, DB, QL, and SC analyzed data; MH, DB, QL, and SC wrote the paper.

### Conflict of interest statement

The authors declare that the research was conducted in the absence of any commercial or financial relationships that could be construed as a potential conflict of interest.
